# Leveraging the private sector for health system development in sub-Saharan Africa

**DOI:** 10.7189/jogh.16.03021

**Published:** 2026-08-21

**Authors:** Faraan O Rahim, Johara Ahmed, Abigail Bekele, Blen Yohannes, Nam T Nguyen, Elshaday Million, Ellen Mkondya-Senkoro, Bernice Brempong, Sangu Delle, Lia Tadesse Gebremedhin

**Affiliations:** 1 Harvard Medical School, Boston, USA; 2 San Jose State University, San Jose, USA; 3 San Francisco State University, San Francisco, USA; 4 Northeastern University, Boston, USA; 5 University of California San Diego, USA; 6 University of Maryland, College Park, USA; 7 Benjamin Mkapa Foundation, Dar es Salaam, Tanzania; 8 Office of the President, Republic of Ghana, Accra, Ghana; 9 CarePoint, Accra, Ghana; Lagos, Nigeria; Cairo, Egypt; 10 Harvard T. H. Chan School of Public Health, Boston, USA

**Keywords:** global health, sub-Saharan Africa, health systems strengthening, private health sector

## Abstract

Development assistance has historically supported major gains in health and health-system development in sub-Saharan Africa, but recent reductions in development assistance have intensified financing and service-delivery pressures across the region. As governments seek more durable and domestically sustained strategies, the private health sector may play a larger role in expanding financing, specialised care, innovation, workforce retention, and public-private partnerships. However, private sector expansion alone is not sufficient and may worsen inequities if it remains disconnected from national financing frameworks, accreditation systems, quality standards, and public accountability. Here, we argue that private providers should be incorporated into well-regulated mixed health systems that align commercial incentives with universal health coverage, equity, and public health goals. Experiences from Ghana and Kenya have shown that integration depends on timely reimbursement, efficient accreditation, and deliberate efforts to reach underserved populations. Private-sector engagement should therefore complement, not substitute for, strong public health systems.

Over the past three decades, development assistance has supported substantial progress in human health and health system development in sub-Saharan Africa (SSA). Between 1995 and 2014, SSA received an estimated USD 114.3 billion in health development assistance, primarily from the USA and Europe [1]. US Agency for International Development (USAID), the largest development assistance agency, has prevented more than 90 million deaths and reduced all-age mortality by 15% globally, with the largest gains in SSA [2]. President's Emergency Plan for AIDS Relief, an HIV/AIDS-focused development assistance initiative, is credited with saving more than 25 million lives and strengthening laboratory and supply chain systems across SSA [3,4]. Multilateral initiatives such as the Global Fund and the World Health Organization (WHO) have also played key roles in financing essential services, expanding the health workforce, and improving disease surveillance across the region [5].

Despite these strides, recent geopolitical changes have upended development aid for health in SSA. Since January 2025, the USA has dismantled USAID and withdrawn from WHO, significantly reducing the flow of development assistance to SSA’s health systems [6,7]. Other international donors have also reduced their contributions, including the UK, France, and the Netherlands, leading to decreased funding of key initiatives for epidemic surveillance, maternal and child health, and non-communicable diseases [6]. Without alternative financing strategies, these cuts threaten to reverse progress in epidemic control, maternal and child health, and broader health system capacity.

Reductions in development assistance and consequent threats to health gains in SSA call for a new approach to advancing health systems in the region. Here, we draw on peer-reviewed health-systems literature and country-level examples to examine the potential role of private-sector engagement across the region’s heterogeneous health systems. We argue that a strengthened, well-regulated private sector could help address gaps in health financing and offer a longer-term, domestically anchored solution. Private providers already deliver a substantial share of care in SSA, estimated at 40% of all healthcare services in the WHO African region [8].

The private sector, however, includes varied stakeholders, such as tertiary hospitals, faith-based and informal providers, drug manufacturers, telemedicine enterprises, and public-private partnerships, each with unique incentives and implications [9]. The contribution of the private sector depends not only on its expansion, but also on the government’s capacity to incorporate certain actors into healthcare funding, accreditation, quality control, and equity goals. In settings with weak government oversight, mixed health systems may experience Nishtar’s ‘mixed health systems syndrome’ whereby fragmented financing and insufficient regulation lead to poor access, low-quality services, and inequity [10]. Consistent with this expectation, Basu *et al.* found that private-sector services were no more efficient, accountable, or effective than public-sector services, and often performed worse regarding equity [11]. Consequently, private-sector development should be assessed in the context of achieving equitable universal health coverage.

A stronger private health sector in SSA has the potential to generate domestic revenue while also addressing long-standing challenges of outbound medical tourism. In oncology, for instance, limited cancer-care infrastructure and workforce capacity have contributed to referrals abroad. In a survey of clinicians from 17 African countries, 75% reported having referred patients abroad for cancer treatment [12]. Additionally, Afreximbank estimates that Africans spend USD 6–10 billion annually seeking treatment abroad, suggesting that expanded access to specialised care within the continent could retain a portion of this healthcare spending [13]. Investing in private health providers may help establish regional centres of excellence that retain some patients on the continent for care, generating income that can be reinvested in health system strengthening and broader economic growth in SSA. Private providers are also well positioned to participate in public–private partnerships (PPPs) that complement public health facilities. However, the drivers of outbound medical travel extend beyond the availability of private facilities and include limited infrastructure, specialist-workforce shortages, and broader system constraints. Private-sector investment must therefore be accompanied by broader health-system strengthening if it is to reduce reliance on care abroad.

Stronger investment in SSA’s private health sector could help to address the ‘brain drain’ of medical professionals. The region loses thousands of physicians each year to emigration, often driven by higher salaries and better working conditions abroad [14]. A better-financed private sector can help counter this trend by offering attractive career paths, improved working conditions, and competitive compensation for providers within the region. Although higher compensation may improve retention, physician migration also reflects limited postgraduate training opportunities, constrained professional development pathways, and governance instability [14]. Private-sector investment should therefore complement, rather than replace, public investment in training, research, and academic medicine. However, this expansion may also draw clinicians out of already understaffed public facilities, producing internal brain drain. For example, in Mozambique, internal migration from the public sector exceeded emigration as a source of physician loss, and dual practice can act as a stepping stone out of public service rather than an anchor within it [15,16]. Retention strategies must therefore safeguard public-sector staffing.

Investment in private-sector innovation could also strengthen digital health, supply chains, and regionally anchored pharmaceutical and vaccine production [17,18]. In many SSA countries, governments and public-health partners can use regulatory coordination, interoperability standards, and strategic procurement to scale locally relevant technologies, strengthen manufacturing capacity, and reduce dependence on external suppliers.

Despite the promise of SSA’s private health sector, structural constraints currently limit its growth. Delayed insurance reimbursements, high cost of capital, currency volatility, and limited access to long-term financing for infrastructure expansion materially affect the ability of private providers to scale high-quality tertiary and specialised services [8]. As a result, the private healthcare landscape in SSA is largely stratified, comprising a mix of high-quality institutions, facilities with infrastructure gaps, and low-cost providers of variable quality [19]. Meanwhile, many private health services disproportionately serve urban, higher-income populations and remain unaffordable among underserved populations due to higher costs and out-of-pocket payment structures [9]. Many countries in SSA also lack mechanisms needed to integrate private providers into national health strategies, including purchasing and contracting arrangements, pooled financing models, and strong implementing partners, which ultimately impedes the formation of PPPs [20].

As aid diminishes, mobilising alternative sources of health financing will become necessary. Private financing for healthcare in SSA has expanded slowly and remains limited, in part because health funding in the region has historically relied on development aid rather than private investment. To prevent private investment from widening inequities, governments should require publicly supported private providers to deliver affordable, high-quality services and improve access for underserved populations.

Structural barriers to private health sector growth must be addressed in tandem with stronger regulatory and integration frameworks. Private facilities should be formally incorporated into national health strategies and linked to public financing mechanisms, enabling them to function as credible, sustainable partners within SSA health systems. Well-designed PPPs can channel revenues from the private sector to subsidise care for underserved populations in public facilities [21]. These arrangements should be supported by accreditation systems, payment reforms, and accountability mechanisms to ensure equitable service delivery. Achieving these goals will require governments to operationalise accreditation, reimbursement, and accountability through adequately staffed regulatory agencies, enforceable contracts, and routine monitoring of quality and equity outcomes. Without these implementation capacities, private-sector expansion risks reinforcing rather than resolving inequities within mixed health systems [10].

Early experiences with national health insurance in Ghana and Kenya illustrate both the potential and the pitfalls of public–private health sector integration. In Ghana, private facilities constitute a substantial share of the National Health Insurance Scheme (NHIS) network, yet only about 31% of facilities operate at optimal technical efficiency, and most accredited private providers achieve only average quality grades [22]. In Kenya, the National Hospital Insurance Fund provides 60-minute spatial access to 81.4% of the population overall, but this masks stark regional inequalities, with access falling to 28.1% in marginalised counties such as Wajir [23]. Qualitative evidence from 79 private facilities in Ghana and Kenya further found that delayed reimbursement constrained participation in Ghana’s NHIS, while lengthy accreditation processes discouraged participation in Kenya’s NHIF [24]. Together, these experiences underscore that while national insurance and PPPs can integrate the private sector, their viability depends on timely reimbursement and deliberate efforts to reach underserved regions.

Private sector expansion in SSA should not be a substitute for a strong public health system. Rather, its value lies in whether it can be integrated into an equity-focused health system, where public and private providers are held to shared standards of access and accountability. As development assistance declines, intensifying financing and service-delivery pressures, governments should strengthen transparent accreditation, timely reimbursement, and PPP frameworks that hold private providers accountable to public health goals. With this approach, private-sector engagement can complement public investment and expand capacity.

## Data Availability

**Data availability:** Not applicable.
